# Carotid Plaque Age Is a Feature of Plaque Stability Inversely Related to Levels of Plasma Insulin

**DOI:** 10.1371/journal.pone.0018248

**Published:** 2011-04-07

**Authors:** Sara Hägg, Mehran Salehpour, Peri Noori, Jesper Lundström, Göran Possnert, Rabbe Takolander, Peter Konrad, Stefan Rosfors, Arno Ruusalepp, Josefin Skogsberg, Jesper Tegnér, Johan Björkegren

**Affiliations:** 1 The Cardiovascular Genomics Group, Department of Medical Biochemistry and Biophysics, Solna, Karolinska Institutet, Stockholm, Sweden; 2 Clinical Gene Networks AB, Stockholm, Sweden; 3 Ion Physics, Department of Physics and Astronomy, Uppsala University, Uppsala, Sweden; 4 The Computational Medicine Group, Department of Medicine, Solna, Center for Molecular Medicine, Karolinska Institutet, Stockholm, Sweden; 5 Department of Surgery, Södersjukhuset, Karolinska Institutet, Stockholm, Sweden; 6 Department of Clinical Physiology, Södersjukhuset, Karolinska Institutet, Stockholm, Sweden; 7 Department of Cardiac Surgery, Tartu University Hospital, Tartu, Estonia; Universitätsklinikum Schleswig-Holstein - Campus Luebeck, Germany

## Abstract

**Background:**

The stability of atherosclerotic plaques determines the risk for rupture, which may lead to thrombus formation and potentially severe clinical complications such as myocardial infarction and stroke. Although the rate of plaque formation may be important for plaque stability, this process is not well understood. We took advantage of the atmospheric ^14^C-declination curve (a result of the atomic bomb tests in the 1950s and 1960s) to determine the average biological age of carotid plaques.

**Methodology/Principal Finding:**

The cores of carotid plaques were dissected from 29 well-characterized, symptomatic patients with carotid stenosis and analyzed for ^14^C content by accelerator mass spectrometry. The average plaque age (i.e. formation time) was 9.6±3.3 years. All but two plaques had formed within 5–15 years before surgery. Plaque age was not associated with the chronological ages of the patients but was inversely related to plasma insulin levels (p = 0.0014). Most plaques were echo-lucent rather than echo-rich (2.24±0.97, range 1–5). However, plaques in the lowest tercile of plaque age (most recently formed) were characterized by further instability with a higher content of lipids and macrophages (67.8±12.4 vs. 50.4±6.2, p = 0.00005; 57.6±26.1 vs. 39.8±25.7, p<0.0005, respectively), less collagen (45.3±6.1 vs. 51.1±9.8, p<0.05), and fewer smooth muscle cells (130±31 vs. 141±21, p<0.05) than plaques in the highest tercile. Microarray analysis of plaques in the lowest tercile also showed increased activity of genes involved in immune responses and oxidative phosphorylation.

**Conclusions/Significance:**

Our results show, for the first time, that plaque age, as judge by relative incorporation of ^14^C, can improve our understanding of carotid plaque stability and therefore risk for clinical complications. Our results also suggest that levels of plasma insulin might be involved in determining carotid plaque age.

## Introduction

Atherosclerosis is believed to be a life-long chronic inflammatory disease initiated at locations of turbulent blood flow, where lipid-laden macrophages accumulate in the arterial wall and eventually form mature plaques [1]. The cellular content and relative amount of fat and collagen in the plaques is thought to determine the risk of spontaneous plaque rupture [2], [3]. In the coronary arteries, plaque rupture can lead to thrombus formation and myocardial infarction. A thrombus formed after carotid plaque rupture can cause transient ischemic attacks (TIAs) or stroke. In humans, the progression of carotid atherosclerosis (i.e., plaque growth) can be estimated by the intima-media thickness (IMT) assessed by noninvasive B-mode ultrasound [4]. IMT has also been shown to predict future cardiovascular events, such as TIA, stroke, and even myocardial infarction [5], [6]. However, the role of IMT as a marker of plaque stability (i.e., risk of rupture) has been questioned. For instance, young individuals with increased IMT have a lower absolute risk for clinical events [6]. Other ultrasound characteristics of carotid plaques have been sought to assess carotid plaque stability. On such characteristic is the degree of echogenicity [7]. Echo-lucent plaques are lipid- and macrophage-rich, signs of instability that are associated with a greater risk of clinical symptoms than echo-rich plaques, which are fibrotic and calcified, hallmarks of stable plaques [7]. Echo-lucent plaques are also associated with increased oxidative stress and inflammation independent of IMT [8].

Since extensive nuclear bomb tests in the 1950s and 1960s leading to a rapid increase in atmospheric concentrations of carbon-14 (^14^C), the ^14^C concentration has steadily been declining [9]. This curve of declination can now be used to our advantage allowing dating of biological materials synthesized over the last five to six decades. Through isolation of ^14^C from any biological sample using accelerator mass spectrometry (AMS), it is possible to determine its relative content of ^14^C that can be mapped on to the declination curve determining the year of synthesis.

The role of tissue age for physiological functions and pathological dysfunctions, is not very well understood. A reason for overseeing age aspect of biology is most likely the lack of reliable technologies to estimate the average age of cells and tissues. However, as a result of recent developments it is now possible to ^14^C date small (µg) amounts of biological samples [10], [11]. These improvements have already lead to several major contributions to science improving our understanding of fat cell and cardiomyocyte turnover with consequences for risk of diseases in these tissues [12],[13]. In addition, for certain types of diseases, like atherosclerosis and cancer tumors that also are containing necrotic biological material processed both within and outside cells and thus remaining in the lesions across many cell generations, ^14^C dating of the entire disease lesion is likely to provide a better understanding of the development of these diseases over time and therefore risk for clinical implications than ^14^C dating DNA alone [12], [13].

In the current study, we determined the average age of carotid plaques using AMS to estimate the relative incorporation of ^14^C in the core of plaques from 29 well-characterized symptomatic patients with carotid stenosis. We hypothesized that carotid plaque age (*i.e.* the average formation time of the plaques) could be an important feature of plaque stability (as determined by immunohistochemistry (IHC)) and, as such, indicate the risk of clinical events. We also sought associations between average plaque age and the clinical phenotypes of the patients as well as gene expression phenotypes of the plaques. The latter being assessed by using Affymetrix GeneChip microarrays. We found that a low plaque age, (reflecting a shorter plaque formation time) was characterized by IHC features of unstable plaques. A lower plaque age was also associated with higher levels of plasma insulin and of gene activity related to oxidative phosphorylation and immune responses.

## Results

### The biological age of carotid plaques determined by ^14^C dating

**Figure 1:**
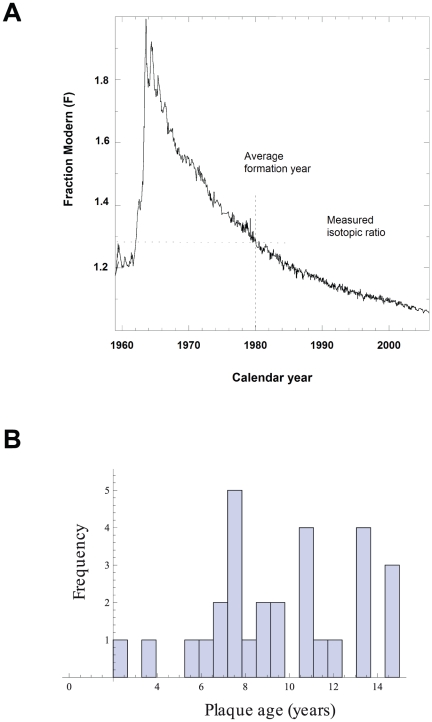
The bomb curve and histogram of the carotid plaque age distribution. (**A**) The bomb curve. Carotid plaque samples were ^14^C dated using AMS. The cellular birth dates can be inferred by determining the time at which ^14^C concentration of the sample corresponded to the atmospheric concentration, using the Levin data as reference bomb curve. (**B**) Plaque age distribution in all carotid stenosis patients (n = 29). The interval is from 2–14 years, and all but two patients had plaque ages within a 10-year interval.

The cores of plaques isolated from the common carotid arteries of symptomatic patients (e.g., TIA) [14] were ^14^C dated. The principle of ^14^C dating of biological samples is shown in Fig. 1A. In brief, the levels of atmospheric ^14^C have been gradually declining since the nuclear bomb tests in the 1950s and 1960s. The curve of declination can be used to measure the time of biosynthesis and thus the average biological age of any living matter formed during this period (i.e. the formation time). This includes different components of the body as well as disease processes, like plaque formation. The average age of the plaque cores in our study was 9.6±3.3 years (n = 29).

The patient-to-patient variation of carotid core formation, reflected by the standard deviation of 3.3 years, was surprisingly low given the heterogeneity of the biological materials (e.g., lipids and collagen) and cell types in the core, suggesting a more regulated and controlled formation than expected. None of the samples were older than 15 years and in >93% (27/29) of the patients, the cores were formed 5 to 15 years before surgery (Fig. 1B). Two patients had a plaque age of 2 and 4 years, respectively. ^14^C dating of carotid plaque cores divided into two halves isolated from two additional patients showed a low intra-sample variation; the average age differences between the two core halves in these two patients were 0.9 year and 0.6 years, respectively.

### Echogenicity and IHC characteristics of the carotid plaques

The average echogenicity of the carotid plaques as determined by Gray-Weale scale (2.24±0.97, range 1–5, with 1 being echo-lucent and 5 echo-rich, Table 1) suggested that all carotid plaques were mostly echo-lucent, thus rich in lipids and macrophages. This is consistent with the clinical symptoms observed in most patients of carotid plaque instability (n = 25/29, Table S12).

**Table 1:** Clinical characteristics of all patients and in terciles of these patients.

Characteristics	All patients (n = 29)	High plaque age (n = 10)*	Intermediate plaque age (n = 9)*	p	Low plaque age (n = 10)*	*p*
Plaque age (years)	9.61±3.31	13.23±1.25	9.48±1.19	***	6.11±1.73	***
Plaque start age (years)	58±10	56±8	63±9		57±12	
Age (years)	68±10	69±8	72±9		63±12	
Intima-media thickness (mm)	1.25±0.23	1.44±0.30	1.13±0.17		1.19±0.09	
Gray-Weale scale (1–5)	2.24±0.97	2.25±1.49	2.00±0.76		2.44±0.53	
Grayscale Median scale	25±17	24±19	20±10		30±19	
Male	55 (16)	56 (5)	63 (5)		67 (6)	
Body mass index (kg/m^2^)	25.1±3.6	24.2±4.1	25.8±2.7		25.5±3.9	
Waist-to-hip ratio	0.90±0.06	0.87±0.04	0.92±0.05	*	0.92±0.07	
Blood pressure (mm Hg)						
Systolic	154±19	155±11	152±24		155±15	
Diastolic	78±8	76±8	79±10		79±9	
Insulin (pmol/L)	45±18	31±11	47±15	*	57±18	**
Proinsulin pmol/L	4.9±2.6	5.2±3.1	4.3±2.5		5.2±2.2	
HbA1c (%)	4.9±0.5	5.1±0.6	4.6±0.4		5.0±0.4	
C-reactive protein (mg/L)	10.5±16.1	7.70±10.27	9.40±14.84		14.25±21.99	
Cholesterol (mmol/L)						
Total	4.64±1.12	4.79±1.32	4.96±1.26		4.21±0.69	
VLDL	0.25±0.17	0.28±0.22	0.21±0.10		0.26±0.16	
LDL	2.52±0.87	2.60±0.96	2.81±1.00		2.19±0.60	
HDL	1.69±0.41	1.75±0.54	1.72±0.34		1.61±0.33	
Triglycerides (mmol/L)						
Total	1.29±0.46	1.30±0.62	1.19±0.32		1.38±0.42	
VLDL	0.85±0.40	0.88±0.60	0.73±0.23		0.92±0.32	
LDL	0.30±0.10	0.28±0.08	0.31±0.09		0.30±0.12	
HDL	0.19±0.04	0.19±0.04	0.19±0.03		0.19±0.05	
Smoking status				NA		NA
Current	7 (2)	20 (2)	0 (0)		0 (0)	
Former ≤2 years	38 (11)	40 (4)	63 (5)		22 (2)	
Former >2 years	28 (8)	0 (0)	25 (2)		67 (6)	
Nonsmoker	21 (6)	40 (4)	13 (1)		11 (1)	
Alcohol consumption (g/week)	102±108	58±53	114±164		142±89	*
Diabetes mellitus	7 (2)	11 (1)	0 (0)		11 (1)	
Insulin-requiring	3 (1)	0 (0)	0 (0)		11 (1)	
Hyperlipidemia	48 (14)	56 (5)	63 (5)		44 (4)	
Statins	59 (17)	44 (4)	57 (4)		100 (9)	
Hypertension	62 (18)	78 (7)	75 (6)		56 (5)	
Beta blocker	38 (11)	44 (4)	50 (4)		33 (3)	

Values are mean ± SD or % (n). HbA1c, glycated hemoglobin; VLDL, very low density lipoprotein; LDL, low density lipoprotein; HDL, high density lipoprotein; NA, not applicable.

*p<0.05,

**p<0.01,

***p<0.001 vs. high plaque age group.

**Figure 2:**
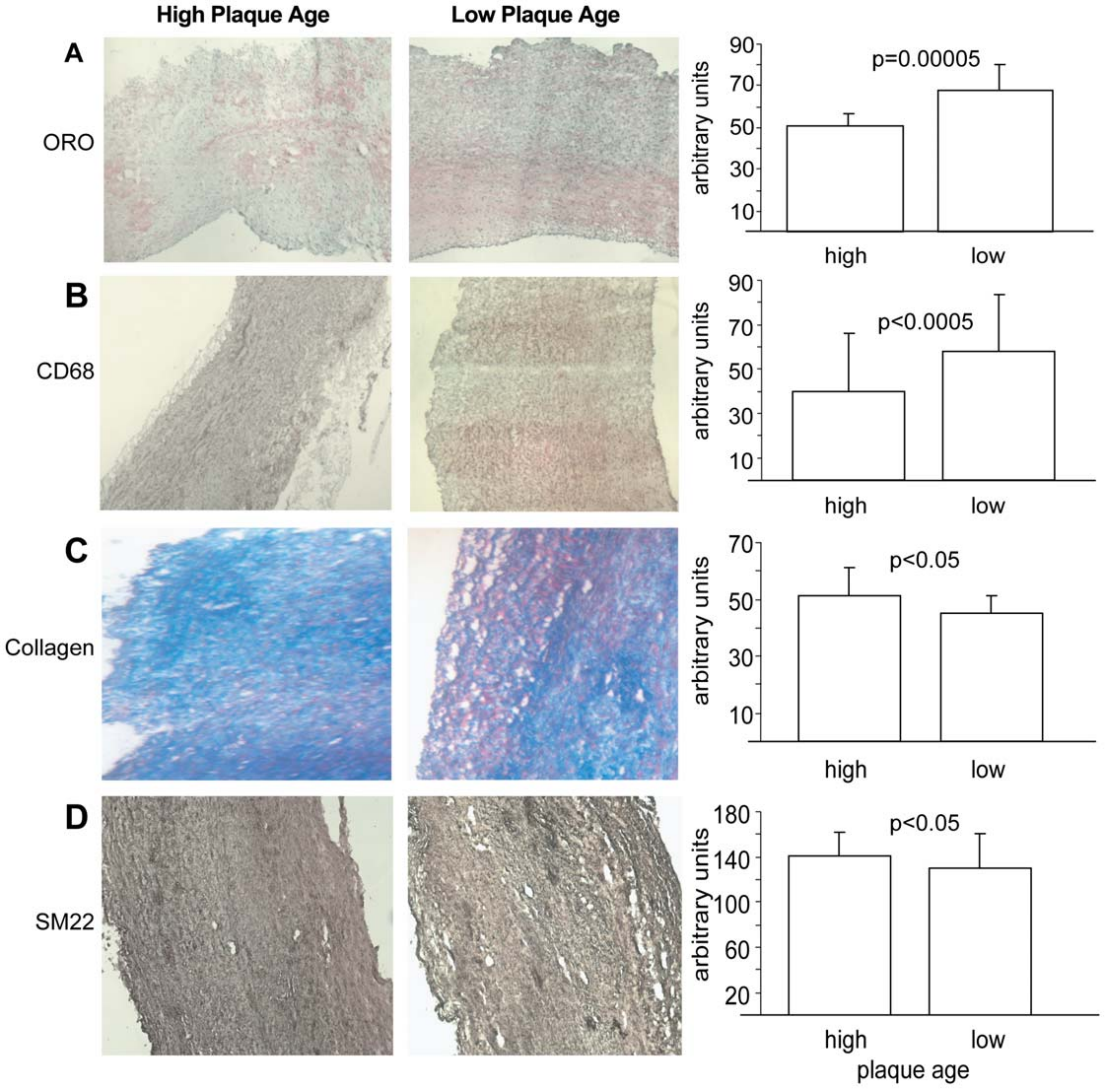
IHC characteristics of plaque age. Representative sections of carotid cores from the terciles of highest (slower formation time) and lowest (faster formation time) average plaque age (see Table 1). The IHC analyses were performed on 7-µm cryosections and stained (**A**) for lipids with Oil-Red-O, (**B**) for macrophages with CD68 antibody, (**C**) for collagen with Masson's trichrome, and (**D**) for smooth muscle cells with SM22 antibody. Bar charts present mean ± SD.

To understand the role, if any, of plaque age for carotid plaque stability, we investigated the IHC characteristics and their associations with plaques of different ages. First, we grouped the patients into terciles by plaque age (Table 1). The difference in plaque age between the two extreme terciles was, as expected, highly significant (Table 1, p = 0.001). Carotid plaque echogenicity did not differ between the terciles of plaque age (Table 2). In contrast, in the lowest tercile of plaque age, the core fat content (determined by Oil-Red-O staining; Fig. 2A) was 35% higher (67.8±12.4 vs. 50.4±6.2, p = 0.00005) and the macrophage content (CD68 staining; Fig. 2B) was 45% higher (57.6±26.1 vs. 39.8±25.7, p<0.0005) than in the highest tercile (n = 6 per tercile). Furthermore, the collagen and SM22 contents were significantly lower (13%, 45.3±6.1 vs. 51.1±9.8, p<0.05, and 8%, 130±31 vs. 141±21, p = 0.05, respectively) than in the highest tercile (Fig. 2C and D). These findings indicate that plaque age distinguishes sublevels of instability of atherosclerotic plaques and that a low plaque age, reflecting faster formation time, is consistent with the IHC phenotype of more unstable plaques.

**Table 2:** Univariate Pearson correlations with plaque age.

Phenotype	Correlation	p-value
Insulin	-0.59	0.0014
logGT	-0.34	0.08
Age	0.34	0.09
logALAT	-0.34	0.09
Hemoglobin	-0.31	0.13
logCreatinine	0.29	0.15
Waist-hip ratio	-0.27	0.18
Diastolic blood pressure	-0.27	0.19
Weight	-0.26	0.20
HDL cholesterol	0.26	0.20
logFibrinogen	0.25	0.22
Plasma cholesterol	0.25	0.22
Waist	-0.24	0.25
logAlcohol consumptionlogVLDL TG	-0.22-0.20	0.310.32
HbA1c	0.20	0.35
LDL cholesterol	0.18	0.37
Height	-0.17	0.42
Body mass index	-0.17	0.42
Thrombocytes	-0.16	0.42
Echogenicity (Gray-Weale)	-0.14	0.50
Plasma TG	-0.12	0.56
Liver-type pyruvate kinase	0.12	0.58
Thyroid-stimulating hormone	-0.10	0.62
Hip	-0.09	0.67
logCRP	-0.07	0.72
Heart rate	0.07	0.72
logVLDL cholesterol	-0.06	0.77
logASAT	-0.05	0.81
Proinsulin	-0.03	0.87
HDL TG	-0.03	0.87
Vmax	0.03	0.91
logLDL TG	-0.01	0.96
Systolic blood pressure	0.00	0.99

GT, gamma-glutamyltransferase; ALAT, alanine aminotransferase; HDL, high density lipoprotein; LDL, low density lipoprotein; VLDL, very low density lipoprotein; TG, triglyceride; HbA1c, glycated hemoglobin; CRP, C-reactive protein; ASAT, aspartate aminotransferase; LDL, low density lipoprotein.

### Clinical characteristics of the patients in relation to plaque age

The carotid stenosis patients (n = 29) were typical for this kind of cohort (Table 1). The average age was 68 years, 55% were males, the average body mass index was just above 25, and blood pressure (38% on beta blockers) and lipid levels (59% on statins) were normal. Only two patients had a diagnosis of diabetes (7%).

Univariate Pearson correlation analyses (Table 2) of plaque age in relation to other patient characteristics showed that the strongest and only significant correlation was an inverse correlation with plasma insulin levels (r = –0.59, p = 0.0014). Surprisingly, the correlation with patient age was weak and statistically insignificant (r = 0.34, p = 0.09). In bivariate correlation analyses (Table S1) that included the four most correlated parameters from the univariate analyses (plasma insulin levels, patient age, alanine transaminase, and gamma glutamyltransferase; Table 2), the inverse correlation with plasma insulin levels was shown to be independent.

### Two-way cluster analysis of global gene expression profiles from the carotid plaques

Instead of examining the activity of individual genes in relation to plaque age, we used two-way clustering [15], [16] to identify groups of functionally related genes of importance for plaque age according to the mRNA signals of the microarrays. Recently, we used this approach to identify a gene module of importance for the extent of coronary atherosclerosis [14]. This approach generates fewer false positives than gene-by-gene analysis; moreover, bioinformatic analyses, such as Gene Ontology and KEGG analyses using DAVID, are more suitable for identifying groups of functionally related genes than a list of top-ranked differentially expressed genes.

**Figure 3:**
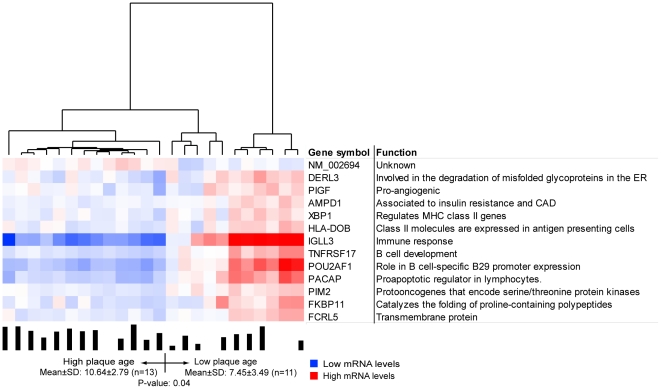
An mRNA cluster segregating patients according to plaque ages. To identify groups of functionally related genes important for plaque age, a two-way clustering approach was used [14]. In the first step, the cluster algorithm is used to determine the total number of functionally related gene groups (i.e., gene clusters) in the carotid plaques calculated from 24 gene expression profiles. Eight gene clusters were identified. In the second step, one cluster (n = 13 RefSeqs/genes) segregated the carotid stenosis patients into two groups that differed significantly in plaque age (p = 0.04), suggesting that these genes could be involved in this plaque formation. Eight of the 13 genes were related to immune or inflammatory processes linked to atherosclerosis.

Gene expression profiles were generated from 25 carotid plaques, 20 of which had been ^14^C dated. In the first step of the two-way clustering analysis, 15,042 RefSeqs/12,621 genes were analyzed in each gene expression profile, which generated a total of eight clusters representing 904 RefSeqs/894 genes. Thus, the carotid lesions harbored eight groups of functionally associated genes active across all patients. In the second step, the patients were clustered according to the RefSeqs signals within each of the eight groups. One gene cluster (n = 13 RefSeqs/genes) segregated the patients into two groups that differed in average plaque age (Fig. 3), suggesting that this cluster is important for plaque formation. Plaque age correlated inversely with the mRNA signals of the 13 genes in this cluster; that is, patients with low plaque ages, and thus faster plaque formation time, had higher expression levels and, as noted earlier, higher plasma insulin levels.

### Bioinformatic analysis of the cluster relating to plaque age

To learn more about genes related to plaque age, we performed a Gene Ontology analysis with DAVID software. Of the 13 cluster genes, five belonged to the biological process immune response (p = 0.001), and an additional three had previously been linked to atherosclerosis [17], [18], [19], [20]. These eight genes were phosphatidylinositol glycan class F (PIGF), with pro-angiogenic properties [20]; adenosine monophosphate deaminase 1 (AMPD1), which is involved in insulin clearance and was previously related to cardiovascular morbidities [17], [19]; tumor necrosis factor receptor superfamily member 17 (TNFRSF17), which is essential for B-cell development; Pou domain class 2 associating factor 1 (POU2AF1), which is critical in B-cell-specific B29 promoter expression; proapoptotic caspase adaptor protein (PACAP), a proapoptotic regulator in lymphocytes [18]; X-box binding protein (XBP1), a transcription factor that regulates major histocompatability complex class II genes [21]; major histocompatibility complex class II (HLA-DOB), which is expressed in antigen-presenting cells; and immunoglobulin lambda-like polypeptide 3 (IGLL3). The remaining five genes (DERL3, PIM2, FKBP11, FCRL5, and NM_002694) have not been extensively studied (total PubMed hits =  155) and have never before been linked to atherosclerotic processes.

### Single-gene analysis using the carotid plaque expression profiles

A number of single-gene analyses were also performed, which in general were less informative (Tables S2, S3, S4, S5, S6, S7, S8, S9, S10, S11). For instance using Spearman rank correlations between plaque age and individual mRNA levels, the 100 most significantly associated genes with plaque age were unrelated to atherosclerosis processes or pathways (Tables S2, S3, S4, S5). In contrast, Spearman rank correlation between mRNA levels and plaque ages performed within each tercile resulted in a group of correlating genes in the low plaque age tercile that was significantly enriched with genes in the oxidative phosphorylation pathway (p = 0.009) (Tables S6 and S9).

## Discussion

In this study, ^14^C dating revealed that the cores of carotid plaques have a much shorter, more uniform biological age, and thus a faster formation time, than would be expected based on the classic understanding of plaque maturation as a lifelong heterogeneous process. Instead, most plaques had formed over a relatively short period of 5 to 10 years (5 to 15 years before clinical symptoms and surgery), and the average subclinical lifespan was ∼9.6 years. The lack of correlation with the age of the patients suggests that carotid plaques could either be formed several times in a lifespan or, alternatively, just once relatively late in life. A faster formation time (i.e., lower biological age) was associated with features of unstable plaques (rich in macrophages and fat, less collagen, and fewer smooth muscle cells). In addition, plaque age was inversely related to plasma insulin levels suggesting that insulin levels may be involved in determining plaque age. Consistent with this notion, genes involved in immune responses and oxidative phosphorylation were more active in plaques with faster formation times.

Our findings suggest that a subtle increase in plasma insulin levels, reflecting mild insulin resistance, increases the pace of plaque formation, possibly by inducing gene activity involving immune responses and oxidative phosphorylation. This would lead to plaques with more macrophages and fat and less collagen, which are more prone to rupture and cause clinical manifestations. Theoretically, improved glucose control might be desirable to prevent diabetes but also to increase plaque stability by prolonging plaque formation time.

Plaque age did not, as might be expected, correlate with chronological age or echogenicity. Thus, other contributing factors may be more important for plaque formation. One obvious possibility is inflammatory processes [8]. Elevated plasma insulin levels were found to be associated with faster formation of the plaques. Thus, mild insulin resistance may be central in increasing the rate of plaque formation, leading to more instable plaques. Indeed, insulin resistance increases levels of proinflammatory cytokines and acute-phase mediators, a state that promotes plaque instability and causes symptomatic carotid atherosclerosis [22], [23], [24]. Our finding that insulin levels are inversely correlated with plaque age may also makes sense considering that elevated plasma insulin levels act through many pathways [24], [25] to increase the growth rate and instability of carotid plaques, consistent with symptomatic disease.

The global analysis of gene expression in the plaque core further supports the notion that mild insulin resistance (only two patients were diagnosed with diabetes) leads to faster plaque formation time, possibly by triggering inflammation in the plaque (and more inflammatory cells, as indicated by IHC analysis of CD68). Indeed, most of the genes we identified as related to low plaque age and faster formation time were associated with inflammatory processes. In addition, the low plaque age samples were significantly enriched with genes of the oxidative phosphorylation pathway, a key driver of oxidative stress, which generates reactive oxygen species, a potent contributor to unstable plaques [26].

Our results suggest that disease tissue formation time is an important but overlooked aspect of disease development. Dating of cardiomyocytes [13], fat cells [12], and neurons [27] has improved our understanding of the etiology of several diseases. However, in contrast to these earlier studies where they ^14^C dated DNA in individual tissue cells [13], [12], [27], we investigated the entire cores of atherosclerotic plaques that besides cells also consists of other types of biological matters that likely is reprocessed both in and outside cells (proteins, fat and necrotic material). For instance, a substantial fraction of the plaque core consists of cholesterol-esters (inside living and necrotic macrophages) originating from the synthesis of fat, cholesterol and lipoprotein particles. The average age of most core material is therefore likely older than its average cell content. Indeed, this seemed to be the case since ^14^C dating the core of carotid plaques indicated an average age of 9.6 years. Or in other words, the ^14^C dating suggests a time of synthesis for most of core matters some 10 years ago. Of course, the biological age of different parts of the core might vary. However, when we additionally ^14^C dated 2 carotid plaque cores divided into 2 random halves (thus 4 independent measurements), the results showed that the variation in biological age between the two halves was less than 1 year (0.9 and 0.6 years, respectively). These results together with relative limited standard variation (SD = 3.3 years) across all core samples suggest a much more coordinated time period for the formation of the plaque cores than perhaps expected, with limited contribution of de-novo synthesized biological matter thereafter.

In accordance, the interpretation of the current ^14^C dating measurements could only with certainty determine the most recent plaque formation time and probably say very little, if anything, about previous reformations and thus plaque regeneration. In fact, it is unclear if the majority of the atherosclerotic plaque core regenerates at all (*i.e.* whether the plaques are reformed several time in a life span or not). Although an average age of 9.6 years suggests that the plaque core very well could regenerate several times in a life span, this will depend on when plaque cores are first formed. Regardless, our study supports the notion that ^14^C dating of entire lesions of diseased tissues (e.g. atherosclerotic plaques, tumors) provides another, but seemingly relevant, type of information than studies performing ^14^C dating of DNA.

In summary, our study of plaque age in relation to clinical and molecular features in carotid stenosis patients revealed that plaque formation time is relevant for plaque stability and therefore risk of clinical events. Interestingly, a recent study of eight carotid stenosis patients used ^14^C dating to investigate formation time of different components of the carotid plaques [28]. Our findings show that ^14^C dating has the potential to deepen our understanding of atherogenesis and its clinical consequences. Additional and larger studies including ^14^C dating of atherosclerotic plaques are warranted.

## Materials and Methods

### Ethics Statement

The study was approved by the Ethics Committee of Karolinska University Hospital and all patients gave written informed consent.

### Study patients and carotid biopsies

Forty-two patients undergoing carotid surgery at Stockholm Söder Hospital were used in this study [14]. At surgery, the core of the carotid plaque was extracted through an incision in the far common arterial wall, embedded in OCT compound (Histolab Products), snap frozen in liquid isopentane and dry ice, and stored at –80°C. Random thirds of the carotid core were used for RNA isolation, IHC, and ^14^C dating. Thirty-nine patients came to a 3-month follow-up visit. Using a standard questionnaire, a research nurse obtained a medical history and lifestyle information (e.g., smoking, alcohol consumption, and physical activity). A physical examination was performed, and venous blood was sampled.

### ^14^C dating of carotid plaques

AMS was used to determine the average formation time of 29 randomly selected cores of the carotid plaques as described [10], [12], [13], [29]. In brief, a 5 MV Pelletron tandem accelerator (NEC) was used to measure the ^14^C:^12^C isotopic ratio. The carotid cores were rinsed in de-ionized water for 3 hours and air-dried. A few milligrams of tissue were placed in a quartz tube with 80 mg of CuO as the oxidizing agent. The tube was flame-sealed and baked at 950°C for 3 hours to produce CO_2_. The gas was cryogenically transferred in vacuum to a vial containing 80 mg of zinc powder as the reducing agent and 2 mg of iron powder as the catalyst. The sealed vial was baked at 530°C for 6 hours, and the graphite produced (∼1 mg) was pressed and loaded into the ion source of the accelerator. Each sample was analyzed during four 5-minute data acquisition periods. After each period, a reference sample (oxalic acid II, NIST) was measured. To correct for fractionation effects, the ^13^C:^12^C ratio of a small part of the CO_2_ gas was measured with an off-line isotope ratio mass spectrometer (Fisons/VG-Isotech 652-Optima). The fractionation-corrected isotopic ratios were presented in fraction modern F^14^C, and the average formation year was extracted [30] based on measurements of CO_2_ variation in northern Europe [9].

### IMT and echogenicity assessed by B-mode ultrasound

Before surgery, carotid arteries were examined with B-mode ultrasound. The far wall of the common carotid artery was used to measure IMT from the endarterectomy side [31]. In addition, The echogenicity of plaques were determined using Gray-Weale [32], [33], [34] and Grayscale Median [35], [36], [37] scales.

### Global gene expression profiling of carotid plaque core

RNA from one third of the carotid plaque core was isolated using Trizol (BRL-Life Technologies), FastPrep (MP Biomedicals), and RNeasy Mini kit (Qiagen) with a DNase 1 treatment step (Qiagen). Sample quality was assessed with an Agilent Bioanalyzer 2100. cRNA yield was assessed with a spectrophotometer (ND-1000, NanoDrop Technologies) before hybridization to HG-U133 Plus 2.0 arrays (Affymetrix). The arrays were processed with a Fluidics Station 450, scanned with a GeneArray Scanner 3000, and analyzed with GeneChip Operational Software 2.0.

### Data pre-processing and clustering

Gene expression values were pre-processed with quantile normalization and the robust multichip average [38]. Of 604,258 perfect-match Affymetrix probe signals, 280,523 could be mapped to 16,685 Entrez genes without cross-hybridizing probes [14].

Coupled two-way clustering [15], [16], [39] was performed to identify small, stable clusters of related signals. In the first step, clusters were defined by superparamagnetic clustering [39], with the absolute value of Spearman rank correlation as a distance measure between genes. Genes that did not belong to a cluster were excluded. In the second step, the identified clusters were related to plaque age by hierarchical clustering [40] of the patients, using Manhattan distance and average linkage as distance measures, based on the mRNA signals in each of the clusters defined in the first step [14].

### Immunohistochemistry

IHC was performed on cryosections (7 µm) from the carotid plaque core. For analyses of CD68 and smooth muscle cells (SM22 alpha), the sections were fixed in acetone. Endogenous peroxidase activity was quenched with 0.3% hydrogen peroxide/0.01% NaN_3_ in water for 10 min, and the sections were incubated with 5% blocking serum. Consecutive sections were incubated with monoclonal mouse anti-human CD68 (Novocastra Laboratories) or polyclonal rabbit anti-human SM22 alpha (transgelin, Abcam) at 4°C overnight. In negative controls, primary antibody was replaced with PBS. After rinsing in Tris-buffered saline, sections were incubated with secondary biotinylated bovine anti-mouse or anti-rabbit (Vector Laboratories). Avidin-biotin peroxidase complexes (Vectastain ABC Elite, Vector Laboratories) were added followed by visualization with diaminobenzidine (Vector Laboratories). All sections were counterstained with Gill hematoxylin (Histolab Products AB). For analysis of collagen, the sections were stained with Masson's trichrome stain (Sigma-Aldrich). Lipid content was analyzed by Oil-Red-O staining, as described [41]. VectaMount (Vector Laboratories) was used as the mounting medium in all experiments. The extent of staining in different sections of the carotid core was assessed with Adobe Photo Shop CS3.

### Statistical analysis

Clinical, IHC, and metabolic characteristics are given as continuous variables with means and standard deviations and as categorical variables with numbers and percentages of subjects. For continuous variables, p-values were calculated with unpaired *t* tests; skewed values were log-transformed. For categorical variables, chi-square or Fisher's exact text (n<5) was used. The correlation analysis was performed with Spearman rank correlation, using p-values from *t* tests after Fisher transformation. Models of interest were identified by multiple step-wise regression analysis. Mathematica 6.0 or SAS 9.0 was used for all calculations.

## Supporting Information

Table S1 Bivariate correlations with top ranked parameters.
(XLS)

Table S2 100 top-ranked genes positively correlated to plaque age.
(XLS)

Table S3 100 top-ranked genes negatively correlated to plaque age.
(XLS)

Table S4 Gene list annotation of Table S2 using DAVID.
(XLS)

Table S5 Gene list annotation of Table S3 using DAVID.
(XLS)

Table S6 100 top-ranked genes correlated to low plaque age.
(XLS)

Table S7 100 top-ranked genes correlated to intermediate plaque age.
(XLS)

Table S8 100 top-ranked genes correlated to high plaque age.
(XLS)

Table S9 Gene list annotation of Table S6 using DAVID.
(XLS)

Table S10 Gene list annotation of Table S7 using DAVID.
(XLS)

Table S11 Gene list annotation of Table S8 using DAVID.
(XLS)

Table S12 Clinical symptoms at entry.
(XLS)
